# Temporal trends (1977-2007) and ethnic inequity in child mortality in rural villages of southern Guinea Bissau

**DOI:** 10.1186/1471-2458-11-683

**Published:** 2011-09-02

**Authors:** Ila Fazzio, Vera Mann, Peter Boone

**Affiliations:** 1 Effective Intervention, Centre for Economic Performance, London School of Economics, Houghton Street, London, UK; 2 Medical Statistics Unit, London School of Hygiene and Tropical Medicine, Keppel Street, London, UK

## Abstract

**Background:**

Guinea Bissau is one of the poorest countries in the world, with one of the highest under-5 mortality rate. Despite its importance for policy planning, data on child mortality are often not available or of poor quality in low-income countries like Guinea Bissau. Our aim in this study was to use the baseline survey to estimate child mortality in rural villages in southern Guinea Bissau for a 30 years period prior to a planned cluster randomised intervention. We aimed to investigate temporal trends with emphasis on historical events and the effect of ethnicity, polygyny and distance to the health centre on child mortality.

**Methods:**

A baseline survey was conducted prior to a planned cluster randomised intervention to estimate child mortality in 241 rural villages in southern Guinea Bissau between 1977 and 2007. Crude child mortality rates were estimated by Kaplan-Meier method from birth history of 7854 women. Cox regression models were used to investigate the effects of birth periods with emphasis on historical events, ethnicity, polygyny and distance to the health centre on child mortality.

**Results:**

High levels of child mortality were found at all ages under five with a significant reduction in child mortality over the time periods of birth except for 1997-2001. That period comprises the 1998/99 civil war interval, when child mortality was 1.5% higher than in the previous period. Children of Balanta ethnic group had higher hazard of dying under five years of age than children from other groups until 2001. Between 2002 and 2007, Fula children showed the highest mortality. Increasing walking distance to the nearest health centre increased the hazard, though not substantially, and polygyny had a negligible and statistically not significant effect on the hazard.

**Conclusion:**

Child mortality is strongly associated with ethnicity and it should be considered in health policy planning. Child mortality, though considerably decreased during the past 30 years, remains high in rural Guinea Bissau. Temporal trends also suggest that civil wars have detrimental effects on child mortality.

**Trial Registration:**

Current Controlled Trials ISRCTN52433336

## Background

Although overall rates of child mortality have been dropping steadily in the last 50 years, these declines have not been homogeneous across different regions and countries. Sub-Saharan Africa still has the highest levels of child mortality in the world, accounting for 49.6% of child deaths in 2010 [1], with a decline that is considered slow and insufficient to achieve the Millennium Development Goal 4 in many countries [1-5]. West and Central Africa show the worst rate of improvement in child survival, with only 18% progress between 1990 and 2008 [2]. Africa is also characterized by a wide variation in mortality levels that has not narrowed since the late 1950s. In the late 1950s, under-five mortality ranged from 113 to 381 deaths per 1000 live births (Mauritius and Sierra Leone), by the late 1990s this gap became even wider from 21 to 334 deaths per 1000 live births (Mauritius and Niger) [3]. In 2009 estimates, sub Saharan Africa encompassed 30 out of 31 countries that still showed U5MR higher than 100 per 1000 live births (with the exception of Afghanistan) [4]. It has been suggested that HIV/AIDS epidemic, economic crisis, political instability, and armed conflicts have played a major role in the poor health performance observed in sub Saharan Africa [5].

Guinea Bissau has the 4^th ^highest rate of U5MR in the world, with 193 per 1000 and an annual rate of reduction of 1.1 [4]. However, the decline in child mortality has been poorly documented due to almost none existing registration of vital events due to political instability, lack of administrative resources and war. Guinea Bissau is one of the poorest countries in the world, with more than two-thirds of its population living below the poverty line and a very weak economy that depends mainly on agriculture (cashew nuts, peanuts and fishing are its major exports). Its history is marked by a long armed struggle for independence from Portugal in 1956-1974, and a civil war (June 1998 and May 1999) between the president (supported by troops from Senegal and Guinea Conakry) and a military Junta [6]. Both wars ruined the economy and social infrastructure and intensified the already widespread poverty. A further coup in September 2003 again disrupted economic activity. To worsen the political and economical instability Guinea Bissau has become a focus point for cocaine trafficking.

Despite the overall increased availability of better quality data documenting country specific mortality trends, high quality data remain scarce in many African countries including Guinea Bissau.

Our aim in this study was to use the baseline survey to estimate child mortality in rural villages in southern Guinea Bissau for a 30 years period prior to a planned cluster randomised intervention. We aimed to investigate temporal trends with emphasis on historical events and the effect of ethnicity, polygyny and distance to the health centre on child mortality.

## Methods

### Data collection and study population

Data were collected at a baseline survey prior to a planned cluster-randomized trial on community health interventions to improve child survival (EPICS - Enabling Parents to Improve Child Survival) [7]. Interventions included child and maternal health education, intensive training and mentoring of village health workers to diagnose and provide first-line treatment for children's diseases within the community, and improved outreach services in the villages randomised to the intervention arm.

**Figure 1:**
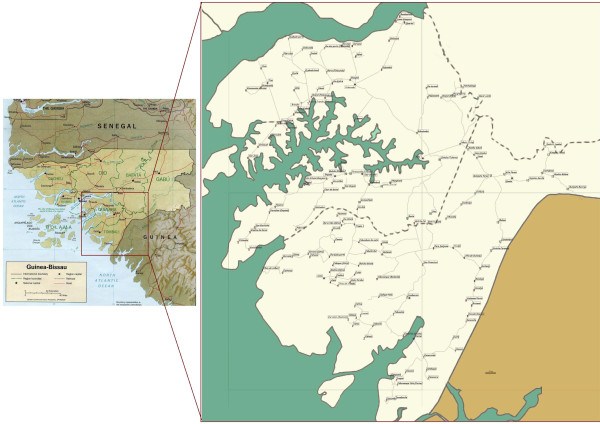
**Map of Guinea Bissau with Quinara and Tombali regions enlarged showing the villages surveyed**.

Guinea Bissau is divided into 8 regions and one autonomous sector. Following the recommendation of the Ministry of Health, villages were selected in the regions of Quinara and Tombali (Figure 1). These regions cover about 20% of the territory and have 11% of the total population. Similarly to the country's ethnic composition, in Quinara and Tombali the population is predominantly Balanta and Fula (though there is also a large number of Beafada). According to the government, these regions had received few health-related projects since the independence. Villages were identified using existing maps and during short fieldwork trips (with local informants' help and a GPS). One hundred and forty six clusters comprising 241 rural villages, about 76% of all rural villages in these regions, were identified to be included in the trial. The identification process is detailed in the protocol [7]. Villages were eligible to be included in the trial if they had an estimated population of 300 to 2000, were not closer than 4 km to another village that was already included, and gave the consent to participate in the trial. If a village had less than 40 houses, up to 4 nearby villages (within 3 km) were grouped to form a cluster to reach the target 40 houses. For villages with more than 52 houses with eligible women, a sub-sample was selected walking away from the centre of the village in all directions so each cluster defined a population of approximately 350 people. Data were collected in each cluster during three days by 5 fieldwork teams (one supervisor and 5 fieldworkers).

A household was registered if it had at least one eligible woman present to be interviewed. A woman was eligible for the trial if she was normally resident in a selected village and a registered house, reported to be between 12 to 49 years old at the time of the visit or the primary care taker of a child (younger than 5 years of age), gave consent and was interviewed at the time of households registration.

The sample used for this article includes only those women who had given birth at least once during the 30 years period before the survey. Child mortality data were obtained from questionnaires and interviews based on DHS model for collecting detailed information on births histories. Births and deaths were dated using events, seasonal calendars, interbirth intervals, and cross-referencing to births of other known-aged children. Given that the main objective of this baseline survey was to collect data on child mortality before planning the trial, questionnaires focused on birth histories and included further information only on distance to health centres, mothers' ethnicity and marriage status.

To attribute ethnicity, each woman was asked to which ethnic group's set of rituals and beliefs she felt the closest affinity. The predominant ethnic groups are: Balantas, Beafadas and Fulas. Other groups include: Nalus, Mandingas, Susso, Bijagós, Pepels, Tandas, Bitcheforés, and Mansuancas. Women were also asked how many co-wives they had and the number of co-wives living with them (referred as polygyny, i.e. marriage system whereby a man can accumulate more than one wife).

The estimated 'walking distance' from each village to the nearest health centre was calculated in hours considering short-cuts and the time to cross the river by canoe.

### Statistical Analyses

All eligible women and their children, reported to be born during the 30 years period preceding the survey were included in the analyses. Distribution of demographic characteristics such as ethnicity, age, and polygyny status of women were tabulated. For continuous variables the mean and standard deviation and for categorical variables numbers belonging to a given category and percentages were calculated.

Crude child mortality rates were estimated using Kaplan-Meier method in 10 yearly periods between 1977 and 1996 and 5 yearly intervals afterwards and also separately for the different ethnic groups. Univariable (simple) and multivariable Cox regression models were used to estimate the effects of birth period, ethnicity, polygyny and the distance to the nearest health centre on child mortality. The multivariable model was built using the variables which had significant effect on mortality on their own. We used robust standard errors to account for the clustering of women in villages and also for the non-independence of children born to the same woman, and performed joint Wald test to test for statistical significance.

### Ethical approval

This study has received ethical approval from the Ministry of Health, Department of Hygiene and Epidemiology, Centre for Coordination of the Research in Guinea Bissau (reference number: 021/2007) as well as from the ethics committee of the London School of Hygiene and Tropical Medicine (reference number: 5173).

## Results

### Descriptive Statistics

Table 1 shows the number of women, their recorded children, and the average number of children per women for each ethnic group (ethnicity is not reported for one woman). On average we interviewed 33 women per village. The mean number of children per woman in this sample is 4.1 (SD = 2.6), with Balanta women showing slightly fewer children per woman (3.7; SD = 2.2) than the others.

**Table 1:** Ethnicity distribution of women and their live born children

Ethnicity	Women	Children	Number of children/woman	
	N	N	mean	SD
Balanta	1658	6059	3.7	2.2
Beafada	2778	11796	4.2	2.7
Fula	1361	5491	4.0	2.5
other	2056	8865	4.3	2.6
missing	1	4	4	-

Total	7854	32215	4.1	2.6

SD standard deviation

Polygyny status per ethnic group is presented in Table 2. Among the married women on average 55% reported to be married monogamously (with no co-wife), 30% reported to have one co-wife and 16% to have two or more co-wives. The numbers of co-wives women reported to actually live with showed very similar results, thus are not separately presented. Co-wife status is not reported for 3 women (1 Beafada, 1 Fula and 1 other) of those for whom we have ethnicity and for a further 15 women, though co-wife status was given, the number of co-wives were not reported (2 Balanta, 7 Beafada, 2 Fula and 4 other). Table 3 describes the age distribution of women at the interview according to their ethnicity. The mean age of women is 30.6 years (SD = 9.4), and this is consistent across all ethnic groups. The mean reported age at first birth is 18.2 years, and Balanta women start having babies one year later than the other groups (Table 4). Age at the first birth was not reported for 4 women.

**Table 2:** Number of co-wives by ethnicity

Ethnicity	Number of co-wives						Total
	none		one		two or more		
	n	%	n	%	n	%	N
Balanta	659	39.8	509	30.7	488	29.5	1656
Beafada	1592	57.5	838	30.3	340	12.3	2770
Fula	768	56.5	414	30.5	176	13.0	1358
other	1274	62.1	566	27.6	211	10.3	2051

Total	4293	54.8	2327	29.7	1217	15.5	7836*

* number of co-wives were not reported for 2 Balanta, 8 Beafada, 3 Fula and 5 other women

**Table 3:** Age distribution of women at interview by ethnicity

		Age at interview (years)			
Ethnicity	n	mean	SD	Inter Quartile range	
Balanta	1658	30.8	9.0	24	37
Beafada	2778	30.3	9.7	23	37
Fula	1361	30.3	9.4	23	37
other	2056	31.1	9.4	23	38

Total	7853	30.6	9.4	23	37

SD standard deviation

**Table 4:** Age distribution of women at first births by ethnicity

		Age at first birth (years)			
Ethnicity	n	mean	SD	range	
Balanta	1657	19.3	3.6	12	40
Beafada	2777	17.9	3.1	10	37
Fula	1361	18.0	3.4	12	37
other	2055	17.9	3.0	12	35

Total	7850*	18.2	3.3	10	40

* age at first birth is missing for 4 women

SD standard deviation

The mean distance to the nearest health centre was reported to be 2.6 hours walking with half of the women reporting less than 2.5 hours (25% of the women reported less than 1.5 hours and 25% of the women reported more than 3.5 hours).

### Child mortality

**Figure 2:**
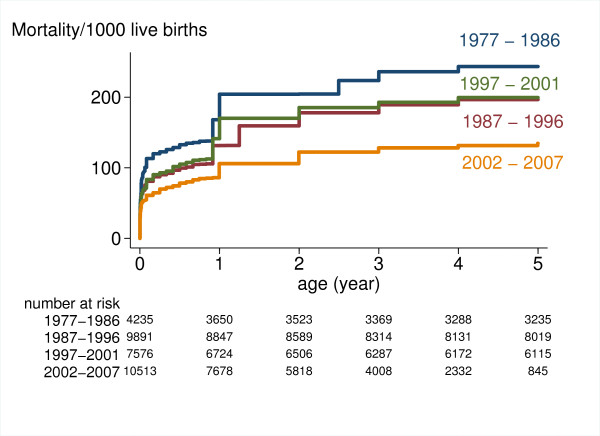
**Age specific mortality rate by period of births**.

Figure 2 presents the age specific mortality rates up to the age of five for birth periods: 1977-1986, 1987-1996, 1997-2001 and 2002-2007. There is a general pattern of decrease in child mortality between a given birth period and the following one, except for the interval between the years of 1997-2001 when neonatal, infant and under-five mortalities were 4.4, 7.6 and 1.5% higher respectively than in the previous period. In the last birth period (2002-2007), the under-five mortality was estimated to be 135 deaths per 1000 live births (95%CI: 127-143 - Table 5).

**Table 5:** Age specific child mortality rates by period of birth

	Birth Period											
	
	l977 - 1986 (n = 4235)			1987 - 1996 (n = 9891)			1997 - 2001 (n = 7576)			2002 - 2007 (n = 10513)		
age	MR	95% CI		MR	95% CI		MR	95% CI		MR	95% CI	
28 days	95	87	105	68	64	74	71	65	77	54	50	59
1 year	168	157	180	131	125	138	141	134	149	106	100	112
2 years	204	192	217	159	152	167	170	162	179	122	116	129
3 years	224	211	237	178	171	186	185	177	194	128	121	135
4 years	236	224	249	189	182	197	193	184	202	132	124	139
5 years	243	231	257	197	189	205	200	191	209	135	127	143

MR Mortality rate: number of death per 1000 live births

CI Confidence Interval

### The effect of ethnicity, polygyny and distance to the nearest health centre

The effects of: birth period, ethnicity, polygyny and distance to the nearest health centre on the hazard to die under five years of age are presented in Table 6 (simple Cox regressions). The results show a statistically significant effect of birth period on child mortality with a general pattern of decrease with an exception for children born in 1997-2001 when hazard is higher than in the previous period.

**Table 6:** Simple Cox regression models with robust standard error to account for clustering of women in villages and more than one child per woman

	n	HR	95% CI		p*
Birth period	32215				< 0.0001
1977 -1986	4235	1	-	-	
1987 -1996	9891	0.78	0.73	0.85	
1997 -2001	7576	0.80	0.73	0.88	
2002 -2007	10513	0.56	0.51	0.62	
					
Ethnicity	32211				0.008
Balanta	6059	1	-	-	
Beafada	11796	0.89	0.81	0.99	
Fula	5491	0.93	0.81	1.05	
other	8865	0.83	0.75	0.93	
					
Has co-wife vs. not	32205				
no	15266	1	-	-	
yes	16939	1.02	0.96	1.08	0.5
					
Number of co-wives	32151				0.09
none	15259	1	-	-	
1	11010	0.99	0.92	1.06	
2+	5882	1.08	1.00	1.18	
					
distance to HC (for 1 hour increase)	32215	1.02	1.00	1.05	0.04

HR hazard ratio

CI Confidence Interval

*p values from Wald test

Although the number of co-wives does not have a statistically significant effect on hazard at 5% (p = 0.09), having 2 or more co-wives increases the hazard by 8% compared to not having co-wife at all. Having co-wife increases the hazard negligibly and the effect is not statistically significant. Longer distance to the nearest health centre also slightly increases the hazard, one more hour to reach the health centre increases the hazard by 2% (p = 0.04).

In the simple regression model with only ethnicity as risk factor in the model, Balanta children have the highest hazard among different ethnic groups (p = 0.008 for heterogeneity). The multiple regression model, built using the variables which had significant effect on mortality on their own, included ethnicity, birth period and distance to the nearest health centre. Longer distance to the nearest health centre increased the hazard the same way as in the simple regression; one more hour to reach the health centre increased the hazard by 2%. This effect was statistically significant at 10% level (p = 0.07). In this model, ethnicity and birth periods, however, violated the proportionality assumption, underlying the Cox regression. This violation disappeared when interaction terms between ethnicity and birth periods were introduced into the model (p < 0.03 for interaction) indicating that the effect of ethnicity is changing by the period of birth. In this final multiple regression model Balanta children have the highest hazard compared to the other ethnic groups until 2002 (Table 7). In the last birth period children of Beafada and other ethnic groups had very similar hazard, and Fula children showed a 21% higher hazard compared to Balanta children.

**Table 7:** Multivariable Cox regression model: effect of ethnicity on mortality by period of birth (adjusted to distance from the closest health centre)

	Born 1977 - 1986			Born 1987 - 1996			Born 1997 - 2001			Born 2002 - 2007		
	N = 4234			N = 9888			N = 7576			N = 10513		
	
ethnicity	HR	95% CI		HR	95% CI		HR	95% CI		HR	95% CI	
Balanta	1	-	-	1	-	-	1	-	-	1	-	-
Beafada	0.86	0.71	1.05	0.81	0.70	0.93	0.97	0.83	1.14	1.00	0.83	1.19
Fula	0.91	0.70	1.19	0.82	0.70	0.97	0.88	0.74	1.05	1.21	0.98	1.49
Other	0.76	0.62	0.94	0.82	0.71	0.95	0.84	0.70	0.99	0.90	0.74	1.10

HR hazard ratio

CI Confidence Interval

## Discussion

This study confirms that despite of substantial decline in the past 30 years, south rural Guinea Bissau still has high levels of child mortality. The under five mortality dropped about 44% in the last 30 years (from 1977-1986 period), with neonatal and infant mortality decreasing 43% and 37% respectively. In the last time period (2002-2007) under our investigation, neonatal death still accounts for 40% of all child mortality with another 38% deaths occurring during the post-neonatal stage below the age of one year, thus future interventions need to focus on these periods of life.

An increase in neonatal, infant and child mortality is observed in the interval of 1997-2001, and we hypothesize that this related to the armed conflict of 1998-1999. Although most of the fighting took place in the capital, up to a third of the country's population was displaced (estimated in 350 000) [8]. No refugee camps were established, but international aid agencies were providing food only to the internally displaced people. Like Aaby *et al. *[8], our results suggest that the hosting population was also very affected by the 1998-1999 war.

The actual detrimental effect of the armed conflict could be even higher if results were presented only for interval of two years that the war lasted. Nielsen *et al. *[9] demonstrated a steep rising in child mortality in a population that fled from Bissau during the war, showing a peek of under-five mortality between June and November 1998, when it was 2.07 times higher than expected. In the same area, the hosting population showed higher levels of malnourishment and child mortality than refugees [8].

For the most recent birth period (between 2002 and 2007) the estimated U5MR was 135 per 1,000 live births (95% CI: 127, 143) (Table 5). This figure is slightly lower than the published estimate for mid 2006 in rural Guinea Bissau that is 179 per 1,000 births [10]. The difference could be explained by the fact that rural data in MICS [10] refers to the whole country and it has been estimated that the mortality level in southern regions is lower than in other areas (east and north) [11].

Many studies have shown that ethnicity affects child mortality [12-17]. Yet, it is difficult to identify the mechanisms that explain observed differences, and these are not necessarily the same for different populations. Several aspects linked to ethnicity may underlie the differences in mortality. Ethnicity may define dissimilarities in socioeconomic characteristics, child care, use of medicines, and health seeking behaviour [12], all aspects that have proven to play crucial roles as determinants of child mortality. Balantas, Fulas and Beafadas have similar economies, based on small-scale agriculture with some cattle husbandry, and it is not clear whether there is a socioeconomic disadvantage by any ethnic group. These ethnic groups, however, have different religions, rituals and settlement patterns. Balantas are Animists, whereas Fulas and Beafadas are Muslims. Their beliefs regarding death are very different, but it is not clear if this results in more or less pragmatic use of western medicines. Although all ethnic groups use traditional doctors and medicines, it has been suggested elsewhere [18] that the use of health services is lower among Balantas.

Overall our data show that Balantas have higher child mortality rate at all ages under five years compared to Fula and Beafada (Table 6). However, as shown in Table 7 this pattern is not consistent over birth periods, with Fula showing higher child mortality in the most recent period. There is no evident reason for why we observe this temporal pattern.

The published literature is also inconclusive about whether Balantas have higher mortality compared to other groups. A longitudinal (1990-1995) study that followed children from rural villages in other regions of Guinea Bissau (Bafatá, Biombo, Cacheu, Gabú and Oio) suggests that Balantas have higher neonatal mortality than other ethnic groups due to lower vaccination coverage and antenatal care [18]. However, a different study showed that in the 1983 measles epidemic, Balantas had a lower risk of dying of measles compared to other ethnic groups [19,20]. The authors suggest that less overcrowding in Balanta houses could be an important factor to explain their observation [19]. In contrary, there is evidence in our study that Balanta women have more co-wives than Fula and Beafada and often live with their co-wives. This would lead to the opposite hypothesis, i.e. Balanta children live in houses with more children. However, with the present data it is not possible to confirm whether there is a significant difference in crowding of children per house. In the capital, Bissau, an ongoing demographic surveillance suggests that Pepel group has higher mortality compared to other ethnic groups [9]. On the other hand, national data from MICS [11] suggests that U5MR are higher among Balanta than Beafada, Fula and other groups.

More research is needed to explain the ethnic differences in mortality, addressing specifically whether these are related to differences in health seeking behaviour rather than just to the physical distance to a health centre, and to investigate the role of household crowding.

Our study showed a slight effect of the distance to the nearest health centre (walking time) on child mortality. Although it is reasonable to expect this association, this could simply indicate the 'level of isolation' of these villages rather than being a measure of the importance of these health centres. A study carried out in Bissau showed that in spite of good health seeking behaviour, the low quality of health services in health centres, especially in recognizing the severity of cases, largely contributed to infant and child mortality [21]. Thus, it is important to assess the quality of services in health centres as well as the care-seeking behaviour. Child mortality is usually lower in urban centres, especially between the ages of one and five years [13]. Even though being close to an urban centre is linked to having a health centre nearer, there are other aspects like schools, quality of housing, socioeconomic status that need to be considered as they might be underlying the effect of health centres proximity.

The fact that we cannot control for maternal education and socioeconomic status might have influenced our results. It has been shown in a national survey that there is a difference in the number of literate women per ethnicity. According to MICS 2006 [11], a higher percentage of Balanta women (23%) were literate compared to Fula and Mandinga women (17%). Socioeconomic status could be also related to 'distance to the health centre' as wealthier families are more likely to live close to facilities.

Child mortality in Africa is still poorly studied, and empirical data during war are often of poor quality or not available. To achieve substantial decline in child mortality it is essential to understand local patterns. This study adds to our knowledge on child mortality in Guinea Bissau, and confirms the increase in child mortality during the civil war period in the southern rural area of the country. It also shows a difference in under-five mortality by the different ethnic groups, although the difference is changing by the period of births.

## Conclusion

Child mortality, though considerably decreased during the past 30 years, remains high in rural villages of Southern Guinea Bissau. It is strongly associated with ethnicity, thus ethnicity should be considered in health policy planning. Temporal trends suggest that civil wars have detrimental effects on child mortality.

## Abbreviations

U5MR: under-five mortality rate; DHS: Demographic Health Survey; MICS: Multiple Indicator Cluster Survey

## Competing interests

The authors declare that they have no competing interests.

## Authors' contributions

All authors contributed to the study design, literature search and revising the manuscript.

IF was responsible for the overall coordination and supervision of data collection, was involved in writing the initial draft and in the interpretation of the results.

VM was responsible for all data analyses, was involved in writing the initial draft and in the interpretation of the results.

PB conceived the study, was supervising the design and execution of the study.

## Pre-publication history

The pre-publication history for this paper can be accessed here:

http://www.biomedcentral.com/1471-2458/11/683/prepub
